# Nutraceuticals, antioxidant pigments, and phytochemicals in the leaves of *Amaranthus spinosus* and *Amaranthus viridis* weedy species

**DOI:** 10.1038/s41598-019-50977-5

**Published:** 2019-12-31

**Authors:** Umakanta Sarker, Shinya Oba

**Affiliations:** 1 Department of Genetics and Plant Breeding, Faculty of Agriculture, Bangabandhu Sheikh Mujibur Rahman Agricultural University, Gazipur, 1706 Bangladesh; 2 Laboratory of Field Science, Faculty of Applied Biological Sciences, Gifu University, Yanagido 1-1, Gifu, Japan

**Keywords:** Biochemical assays, Natural product synthesis

## Abstract

Six selected weedy *Amaranthus* genotypes (three accessions from each species of *A. viridis* and *A. spinosus*) were evaluated in terms of nutrients, minerals, antioxidant constituents and antioxidant activity for the possibilities of weedy species as a vegetable cultivar in a randomized complete block design with three replications. As leafy vegetable, Weedy *Amaranthus* has remarkable protein, dietary fiber, carbohydrates, Ca, K, Mg, P, S, Fe, Mn, Cu, Zn, Na, Mo, B, chlorophylls, β-cyanins, β-xanthins, betalains, β-carotene, vitamin C, TPC, TFC, and TAC (DPPH and ABTS^+^) compared to any cultivated species. The *A. viridis* genotype WAV7 and *A. spinosus* genotype WAS13 had the highest nutrients, pigments, vitamins, phenolics, flavonoids, and antioxidant. Hence, these two weedy accessions could be used as an antioxidant profile enriched cultivar with high nutritional and antioxidant activity. Pigments, β-carotene, vitamin C, phenolics, and flavonoids had strong antioxidant activity and played a vital role in the antioxidant activity of weedy *Amaranthus* genotypes. Weedy species are an excellent source of phenolics, flavonoids, and antioxidants that have many pharmacological and medicinal effects of their traditional applications and detoxify ROS and offered huge prospects for feeding the antioxidant-deficient community to cope with the hidden hunger and attaining nutritional and antioxidant sufficiency.

## Introduction

The family Amaranthaceae consists of 70 *Amaranthus* species of which 17 produce edible leaves and 3 produce food grains^1^. *Amaranthus* species are C_4_ plants with rapidly grown vegetables, ornamental, and grains plants. It is widely distributed and cultivated in Asia, Africa, America, Australia, and Europe. Leaves and succulent stems of *Amaranthus* are inexpensive and excellent sources of protein with essential amino acids lysine and methionine, carotenoids, ascorbic acid, dietary fiber, and essential minerals, such as calcium, magnesium, potassium, phosphorus, iron, zinc, copper, and manganese^2–8^. Some genera of this family are widely used as traditional medicinal plants for remedy of viral diseases, malarial, diabetic, bacterial, helminthic infections and as snake antidote^9–11^. Besides these, it is also an excellent and unique source of antioxidant leaf pigments, such as β-cyanin, β-xanthin, betalain, and a source of other pigments, such as carotenoids, anthocyanin, and chlorophylls^12,13^, and antioxidant phytochemicals, such as β-carotene, vitamin C, phenolics, and flavonoids^14^. Most of these compounds are natural antioxidants and detoxify ROS in the human body, hence, it had great importance for the food industry^15,16^. β-Cyanin, β-xanthin, betalain, carotenoids, and amaranthine pigments have important free radical-scavenging activity^17^. It has wide adaptability to different abiotic stresses like drought^18–21^ and salinity^22–24^ and versatile uses.

Weedy amaranth *(A. spinosus* and *A. viridis*) originates probably from lowland of South and Central America. At present, it is wide spreads over the tropical and subtropical regions, including tropical Africa, South East Asia, Americas as well as temperate Europe. In tropical Africa and elsewhere weedy amaranth leaves and young plants are collected for sale on markets for home consumption as a cooked, steamed or fried vegetable, especially during periods of drought. It tolerates drought, responds to high levels of available nutrients, and adapted to a harsh environment through rapid stem elongation^25–27^.

Weedy amaranth *(A. spinosus* and *A. viridis*) possesses analgesic and antipyretic properties and is used for the treatment of pain and fever as traditional medicine. *A. viridis* weedy amaranth used as antioxidant, antimicrobial, hepatoprotective, anti-nociceptive, anti-inflammatory, hypolipidemic, antihyperglycemic, anthelmintic, anti-phytopathogenic, and antidiabetic activity^28^. Both weedy species have numerous medicinal uses like astringent, diaphoretic, diuretic, emollient, febrifuge, galactogogue, gonorrhea, eczema, burns, wounds, boils, earache, haemorroids, bronchitis. sudorific, antidote to snake poison, menorrhagia. internal bleeding, diarrhea, stomach disorders, ulcerated mouths, nosebleeds, wounds and dysentery^25–27,29^.

In Bangladesh, there are a lot of weedy amaranths *(A. spinosus* and *A. viridis*) grown in the roadside, fallow land and as a weed in the crop field. It is a very popular leafy vegetable and becoming increasingly popular due to its test, flavor, and color. People harvest it and sell in the market as a leafy vegetable. However, no research has been carried out to evaluate the nutritional components, antioxidant phytochemicals and their antioxidant capacity in the leaves of *A. spinosus* and *A. viridis* weedy species. Stinzing *et al*.^30^ reported betacyanins and phenolic compounds in *A. spinosus* stem. But the literature of other cultivated amaranth has shown that leaves contain several times higher nutritional components, antioxidant phytochemicals and their antioxidant capacity than stem^15,31^. Therefore, the present study was undertaken to evaluate the possibility of *A. spinosus* and *A. viridis* weedy species as a leafy vegetable in terms of nutritional components, antioxidant phytochemicals and their antioxidant capacity for achieving nutritional and antioxidant sufficiency in our daily diet.

## Results and Discussion

The analysis of variance revealed that all the studied traits differed significantly in terms of the genotypes (Tables 1, 2, 3, 4, 5). %CV and Mean performance of proximate, mineral compositions, antioxidant leaf pigments, vitamins, TPC, TFC, TAC (DPPH) and TAC (ABTS^+^) in selected six *A. viridis* and *A. spinosus* genotypes are presented in Tables 1, 2, 3, 4, 5.

**Table 1:** Proximate compositions (g 100 g^−1^ fresh weight) and dietary fiber (g 100 g^−1^ FW) of six selected genotypes of *A. viridis* and *A. spinosus* weedy species.

Genotypes	Moisture (g 100 g^−1^)	Protein (g 100 g^−1^)	Fat (g 100 g^−1^)	Carbohydrates (g 100 g^−1^)	Energy (Kcal)	Ash (g 100 g^−1^)	Dietary fiber (g 100 g^−1^ FW)
***A. viridis***							
WAV4	80.35 ± 1.14 f	4.12 ± 0.05b	0.35 ± 0.01e	8.67 ± 0.07b	35.29 ± 0.33a	6.86 ± 0.02a	9.38 ± 0.35b
WAV7	82.28 ± 1.26d	4.52 ± 0.04b	0.42 ± 0.03d	6.31 ± 0.06c	36.72 ± 0.37a	6.75 ± 0.03a	9.17 ± 0.37b
WAV9	81.54 ± 1.18e	4.26 ± 0.06b	0.28 ± 0.04 f	9.03 ± 0.05a	34.98 ± 0.62b	5.43 ± 0.04b	9.26 ± 0.45b
***A. spinosus***							
WAS11	86.26 ± 1.82a	5.54 ± 0.03a	0.51 ± 0.01b	2.33 ± 0.06e	28.45 ± 0.44c	5.62 ± 0.02b	10.65 ± 0.65a
WAS13	84.47 ± 1.34c	5.78 ± 0.06a	0.63 ± 0.02a	4.41 ± 0.03d	27.89 ± 0.46d	5.18 ± 0.04b	11.24 ± 0.72a
WAS15	85.42 ± 1.55b	5.28 ± 0.05a	0.47 ± 0.03c	4.16 ± 0.05d	29.56 ± 0.52c	5.09 ± 0.03b	10.58 ± 0.58a
F values	**	**	**	**	**	**	**
CV%	2.25	1.16	0.18	0.57	0.78	0.46	0.25

CV, Coefficient of variation; n = 3; In a column, mean values with different letters are differed significantly by Duncan Multiple Range Test (P < 0.01).

**Table 2:** Mineral compositions (Macroelements mg g^−1^ FW) of six selected genotypes of *A. viridis* and *A. spinosus* weedy species.

Genotypes	Macroelements (mg g^−1^ FW)				
	K	Ca	Mg	P	S
***A. viridis***					
WAV4	6.61 ± 0.02ab	2.56 ± 0.06ab	3.53 ± 0.03a	0.86 ± 0.001b	1.42 ± 0.06c
WAV7	7.22 ± 0.05a	2.46 ± 0.05ab	3.65 ± 0.07a	0.94 ± 0.002a	1.52 ± 0.05b
WAV9	6.98 ± 0.04a	2.84 ± 0.03a	3.78 ± 0.05a	0.79 ± 0.002b	1.66 ± 0.05a
***A. spinosus***					
WAS11	6.45 ± 0.06ab	2.68 ± 0.04a	2.88 ± 0.03ab	0.72 ± 0.003b	1.34 ± 0.03d
WAS13	6.48 ± 0.04ab	2.44 ± 0.05ab	3.02 ± 0.05ab	0.68 ± 0.005b	1.25 ± 0.04e
WAS15	6.82 ± 0.06a	2.71 ± 0.06a	2.97 ± 0.04ab	0.75 ± 0.003b	1.18 ± 0.02e
F values	**	**	**	**	**
CV%	0.14	0.38	0.26	0.03	0.04

CV, Coefficient of variation; K, Potassium; Ca, Calcium, Mg, Magnesium; P, Phosphorus; S, Sulphur; n = 3; In a column, mean values with different letters are differed significantly by Duncan Multiple Range Test (P < 0.01).

**Table 3:** Mineral compositions (Microelements µg g^−1^ FW) of six selected genotypes of *A. viridis* and *A. spinosus* weedy species.

Genotypes	Microelements (µg g^−1^ FW)						
	Fe	Mn	Cu	Zn	Na	B	Mo
***A. viridis***							
WAV4	21.98 ± 0.09a	8.66 ± 0.06b	2.65 ± 0.03b	12.96 ± 0.08b	28.76 ± 0.10c	11.25 ± 0.02b	0.34 ± 0.05c
WAV7	21.94 ± 0.12a	8.72 ± 0.05b	2.84 ± 0.07b	13.55 ± 0.12b	29.38 ± 0.13b	12.42 ± 0.05a	0.38 ± 0.03b
WAV9	22.18 ± 0.11a	8.99 ± 0.07b	3.02 ± 0.02a	14.72 ± 0.13a	30.26 ± 0.08a	12.74 ± 0.03a	0.43 ± 0.04a
***A. spinosus***							
WAS11	14.86 ± 0.08b	9.74 ± 0.05a	1.37 ± 0.03d	11.35 ± 0.11c	24.56 ± 0.04e	6.35 ± 0.08d	0.35 ± 0.04c
WAS13	15.34 ± 0.09b	10.23 ± 0.06a	2.04 ± 0.07c	10.99 ± 0.13c	25.73 ± 0.14d	7.25 ± 0.06c	0.32 ± 0.02d
WAS15	14.78 ± 0.08b	9.86 ± 0.06a	1.68 ± 0.04d	11.64 ± 0.12c	25.66 ± 0.12d	6.96 ± 0.05c	0.35 ± 0.05c
F values	**	**	**	**	**	**	**
CV%	0.25	0.22	0.08	0.12	0.21	0.07	0.04

CV, Coefficient of variation; Fe, Iron; Mn, Manganese; Cu, Copper; Zn, Zinc, Na, Sodium; Mo, Molybdenum; B, Boron; n = 3; In a column, mean values with different letters are differed significantly by Duncan Multiple Range Test (P < 0.01).

**Table 4:** Mean performance of antioxidant leaf pigments of six selected genotypes of *A. viridis* and *A. spinosus* weedy species.

Genotypes	Chlorophyll *a* (μg g^−1^ FW)	Chlorophyll *b* (μg g^−1^ FW)	Chlorophyll *ab* (μg g^−1^ FW)	β-cyanins (ng g^−1^ FW)	β-xanthins (ng g^−1^ FW)	Betalains (ng g^−1^ FW)	Carotenoids (mg 100 g^−1^ FW)
***A. viridis***							
WAV4	288.33 ± 3.15c	135.26 ± 1.78e	423.59 ± 2.36d	276.34 ± 1.67d	255.78 ± 1.96d	532.12 ± 1.85e	86.48 ± 1.24c
WAV7	302.56 ± 1.23a	142.66 ± 1.68c	445.22 ± 1.22a	285.33 ± 1.84a	246.87 ± 1.51e	532.24 ± 1.64e	92.87 ± 1.33a
WAV9	295.47 ± 3.63b	138.55 ± 1.78d	434.02 ± 3.28b	287.56 ± 1.36a	252.37 ± 1.48d	539.93 ± 1.82d	88.29 ± 1.45b
***A. spinosus***							
WAS11	267.85 ± 3.42e	145.76 ± 1.62b	413.61 ± 1.32e	282.84 ± 1.38b	268.47 ± 1.22c	551.31 ± 2.32c	68.52 ± 1.32e
WAS13	282.36 ± 2.32d	152.42 ± 1.62a	434.78 ± 3.27b	278.49 ± 1.52c	275.86 ± 1.32a	554.35 ± 1.66b	69.82 ± 1.22e
WAS15	284.58 ± 3.22d	146.88 ± 1.69b	431.46 ± 3.35c	286.46 ± 1.24a	274.96 ± 1.38b	561.42 ± 1.84a	71.25 ± 1.15d
F values	**	**	**	**	**	**	**
CV%	2.96	1.32	1.58	2.26	2.43	1.17	1.25

CV, Coefficient of variation; n = 3; In a column, mean values with different letters are differed significantly by Duncan Multiple Range Test (P < 0.01).

**Table 5:** Mean performance of β-Carotene, vitamin C, TPC, TFC, TAC (DPPH) and TAC (ABTS^+^) of six selected genotypes of *A. viridis* and *A. spinosus* weedy species.

Genotypes	β-Carotene (mg 100 g^−1^ FW)	Vitamin C (mg 100 g^−1^ FW)	TPC (GAE µg g^−1^ FW)	TFC (RE µg g^−1^ DW)	TAC (DPPH) (TEAC µg g^−1^ DW)	TAC (ABTS^+^) (TEAC µg g^−1^ DW)
***A. viridis***						
WAV4	62.56 ± 0.62b	104.55 ± 0.19b	40.26 ± 0.27c	174.58 ± 0.36c	23.78 ± 0.08e	45.84 ± 0.41c
WAV7	64.22 ± 0.56a	106.64 ± 0.22a	43.24 ± 0.32b	175.64 ± 0.29c	21.96 ± 0.14 f	48.23 ± 0.31b
WAV9	61.87 ± 0.75b	107.45 ± 0.18a	46.72 ± 0.22a	182.46 ± 0.26a	24.65 ± 0.12d	51.23 ± 0.24a
***A. spinosus***						
WAS11	46.76 ± 0.26e	44.62 ± 0.12e	25.45 ± 0.42d	176.46 ± 0.25c	26.45 ± 0.15b	49.67 ± 0.11b
WAS13	48.28 ± 0.28d	48.72 ± 0.08c	24.98 ± 0.34e	182.36 ± 0.23a	27.56 ± 0.14a	52.35 ± 0.24a
WAS15	52.16 ± 0.88c	46.98 ± 0.09d	26.72 ± 0.32d	178.34 ± 0.16b	25.87 ± 0.11c	47.87 ± 0.27bc
F values	**	**	**	**	**	**
CV%	1.12	1.75	1.69	1.41	0.35	0.38

CV, Coefficient of variation; TAC = Total antioxidant capacity, TPC = Total polyphenol content, TFC = Total flavonoid content, n = 3; In a column, mean values with different letters are differed significantly by Duncan Multiple Range Test (P < 0.01).

### Proximate compositions

Proximate compositions of six *A. viridis* and *A. spinosus* genotypes are presented in Table 1. The moisture content of six selected genotypes of two weedy *Amaranthus* species ranged from 81.54 to 86.26 g 100 g^−1^ FW. The highest moisture content was noticed in *A. spinosus* genotype WAS11 (86.26 g 100 g^−1^ FW) followed by *A. spinosus* genotype WAS15 (85.42 g 100 g^−1^ FW) and WAS13 (84.47 g 100 g^−1^ FW). In contrast, the lowest moisture content was recorded in *A. viridis* genotype WAV4 (80.35 g 100 g^−1^ FW). All the genotypes of *A. viridis* such as WAV4, WAV7, and WAV9 exhibited around 18–20% dry matter could be a promising source of dry matter as higher dry matter ensured with lower moisture contents of leaves. The maturity of the two species could have a vital role in the moisture content of leaves. The moisture contents obtained in our study were fully agreed with the reports of Sun *et al*.^32^ in sweet potato leaves.

As leafy vegetables, leaves of *A. viridis* and *A. spinosus* had high protein content with fewer variations which ranged from 4,12 to 5.78 g 100 g^−1^ FW. The highest protein content was observed in *A. spinosus* genotype WAS13 (5.78 g 100 g^−1^ FW) which was statistically similar to *A. spinosus* genotype WAS11 and WAS15. Conversely, the lowest protein content was exhibited in *A. viridis* genotype WAV4. Weedy amaranth (*A. viridis* and *A. spinosus*) genotypes are the sources of protein for vegetarian and poor people of the third world countries. The protein content of *A. viridis* and *A. spinosus* were much higher as compared to amaranth in our earlier study^5^. In this investigation, *A. viridis* and *A. spinosus* genotypes showed low-fat content as a leafy vegetable and could be used as a cholesterol free food. *A. spinosus* genotype WAS13 showed the highest fat content (0.63 g 100 g^−1^ FW) followed by *A. spinosus* genotype WAS11. Whereas, *A. viridis* genotype WAV9 exhibited the lowest fat content (0.28 g 100 g^−1^ FW) with a range of 0.28 to 0.63 g 100 g^−1^ FW. Fats help in the digestion, absorption, and transport of fat-soluble vitamins A, D, E, K and source of omega-3 and omega-6 fatty acids. Sun *et al*.^32^ reported similar results in sweet potato leaves. They revealed that fat involved in the insulation of body organs and the maintenance of body temperature and cell function.

*A. viridis* genotypes had higher carbohydrates content compared to the genotype of *A. spinosus*. Remarkable variations were observed in the carbohydrate content of *A. viridis* and *A. spinosus* genotypes which ranged from 2.33 to 9.30 g 100 g^−1^ FW. *A. viridis* genotype WAV9 exhibited the highest carbohydrates content (9.30 g 100 g^−1^ FW) followed by *A. viridis* genotype WAV4, while *A. spinosus* genotype WAS11 had the lowest carbohydrates content (2.33 g 100 g^−1^ FW). *A. viridis* genotypes had higher energy compared to the genotype of *A spinosus*. The *A. viridis* genotype WAV4 and WAV7 had the highest energy (36.72, 35.29 Kcal 100 g^−1^ FW) followed by *A. viridis* genotype WAV9. On the other hand, *A. spinosus* genotype WAS13 exerted the lowest energy (27.89 Kcal 100 g^−1^ FW). *A. viridis* genotypes had higher ash content compared to the genotype of *A. spinosus*. The highest ash content was observed in the *A. viridis* genotype WAV4 and WAV7 (6.86, 6.75 g 100 g^−1^), while the lowest ash content was recorded in *A. spinosus* genotype WAS13 (5.18 g 100 g^−1^) which was statistically similar to *A. spinosus* genotype WAS11, WAS15, and *A. viridis* genotype WAV9.

*A. spinosus* genotypes had higher dietary fiber content compared to the genotype of *A. viridis*. The dietary fiber content of selected six *A. viridis* and *A. spinosus* genotypes ranged from 9.17 to 11.24 g 100 g^−1^ FW. *A. spinosus* genotype WAS13, WAS11, and WAS15 showed the highest dietary fiber content (11.24, 10.65 and 10.58 g 100 g^−1^ FW). Conversely, *A. viridis* genotype WAV7 had the lowest dietary fiber content (9.17 g 100 g^−1^ FW) which was similar to WAV4 and WAV9. The dietary fiber played a substantial role in palatability, digestibility, and remedy of constipation^2^. Like other leafy vegetables, our study showed that leaves of *A. viridis* and *A. spinosus* genotypes are an excellent source of moisture, protein, dietary fiber and carbohydrates. *A. viridis* genotype had the highest carbohydrates, energy, and ash content, while *A. spinosus* genotype exhibited the highest moisture, protein, fat, and dietary fiber content.

### Mineral compositions

Mineral compositions of *A. viridis* and *A. spinosus* genotypes are presented in Tables 2, 3. In this study, the highest K content was observed in *A. viridis* genotype WAV7 (7.22 16 mg g^−1^ FW) which was statistically similar to *A. viridis* genotype WAV9 (6.98 mg g^−1^ FW) and *A. spinosus* genotype WAS15 (6.82 mg g^−1^ FW) with a range of 6.45 mg g^−1^ to 7.22 mg g^−1^ FW. Whereas, *A. spinosus* genotype WAS11 and WAS13 exhibited the lowest K content (6.45, 6.48 mg g^−1^ FW which was statistically similar to *A. viridis* genotype WAV4. *A. viridis* genotypes had higher K content compared to the genotype of *A. spinosus*, albeit the differences in K content between to weedy species were no pronounced. Albeit there were no prominent variations in Ca content between to weedy species, *A. spinosus* genotypes had higher Ca content compared to the genotype of *A. viridis* with a range of 2.44 to 2.84 mg g^−1^ FW. The highest Ca content (2.84 mg g^−1^) was reported in *A. viridus* genotype WAV9 which was similar to *A. spinosus* genotype WAS12 and WAS11. In contrast, the lowest Ca content (2.44 mg g^−1^) was obtained from *A. spinosus* genotype WAS13. In this investigation, *A. viridis* and *A. spinosus* genotypes had no pronounced variations in terms of Mg content (2.88 to 3.78 mg g^−1^ FW)*. A. viridis* genotype WAV4, WAV7, and WAV9 exhibited the highest Mg content (3.78, 3.65, 3.52 mg g^−1^ FW), while, *A. spinosus* genotype WAS11, WAS13, and WAS15 showed the lowest Mg content (2.88, 3.02 and 2.97 mg g^−1^ FW). Similarly, *A. viridis* and *A. spinosus* genotypes had no pronounced variations in terms of P content (0.68 to 0.94 mg g^−1^ FW)*. A. viridis* genotype WAV7 exhibited the highest P content (0.94 mg g^−1^ FW), while, *A. spinosus* genotype WAS13 showed the lowest P content (0.68 mg g^−1^ FW) which was statistically similar to *A. spinosus* genotype WAS15, WAS11, and *A. viridis* genotype WAV4 and WAV9. S content had significant variations in six *A. viridis* and *A. spinosus* genotypes which ranged from 1.18 to 1.66 mg g^−1^ FW. *A. viridis* genotypes had higher S content compared to the genotype of *A. spinosus*. *A. viridis* genotype WAV9 exhibited the highest S content (1.66 mg g^−1^ FW) followed by WAV7, while, *A. spinosus* genotype WAS15 and WAS13 showed the lowest S content (1.18 and 1.25 mg g^−1^ FW). Our investigation revealed that we found remarkable K (7.22 mg g^−1^), Ca (2.74 mg g^−1^), Mg (3.52 mg g^−1^), P (0.94 mg g^−1^), and S (1.66 mg g^−1^) in *A. viridis* genotype (fresh weight basis). Jimenez-Aguiar and Grusak^33^ reported high K, Ca, Mg, P, and S (fresh weight basis) in different *A. spp*. including *A. viridis* and *A. spinosus*. They also reported that spider flower, black nightshade, spinach, and kale had much lower K, Ca, and Mg content than amaranth. Our studied *A. viridis* and *A. spinosus* genotype had higher K, Ca, Mg, P, and S (fresh weight basis) compared to studied *A. spp* of Jimenez-Aguiar and Grusak^33^. *A. viridis* genotype had the highest K, Ca, Mg, P, and S content than *A. spinosus* genotype.

In this study, iron content showed significant variations in terms of *A. viridis* and *A. spinosus* genotype. The highest Fe content was recorded in *A*. *viridis* WAV9 (22.18 µg g^−1^ FW) and it was statistically similar to *A. viridis* genotype WAV4 and WAV7. On the other hand, *A. spinosus* genotype WAS15 exhibited the lowest iron content which was statistically similar to *A. spinosus* genotype WAS11 and WAS13. *A. viridis* genotype had higher Fe content compared to *A. spinosus* genotype. Our study revealed that significant variations were observed in Mn content of *A. viridis* and *A. spinosus* genotype. *A. spinosus* genotypes exhibited higher Mn content compared to the genotype of *A. viridis*. Manganese content ranged between 8.66 µg g^−1^ FW and 10.23 µg g^−1^ FW. Manganese content was the highest in *A. spinosus* genotype WAS13 (10.23 µg g^−1^ FW), which was statistically similar to WAS11 and WAS15. Whereas the lowest manganese content was observed in *A. viridis* genotype WAV4, WAV7, and WAV9 (8.66, 8.72 and 8.99 µg g^−1^ FW, respectively). *A. viridis* genotypes exhibited higher copper content compared to the genotype of *A. spinosus*. The copper content had significant notable range of variations in the genotypes of *A. viridis* and *A. spinosus* (1.37 to 3.02 µg g^−1^ FW). *A. viridis* genotype WAV9 had the highest copper content (3.02 µg g^−1^ FW) followed by *A. viridis* genotype WAV4 and WAV7, while *A. spinosus* genotype WAS11 showed the lowest Cu content (1.31 µg g^−1^ FW). The zinc content of *A. viridis* and *A. spinosus* genotypes differed significantly 10.99 µg g^−1^ FW in *A. spinosus* genotype WAS13 to 14.72 µg g^−1^ FW in *A. viridis* genotype WAV9. *A. viridis* genotypes exhibited higher Zn content compared to the genotype of *A. spinosus*. Na content showed significant variations in terms of *A. viridis* and *A. spinosus* genotype. The highest Na content was recorded in *A*. *viridis* genotype WAV9 (25.56 µg g^−1^ FW) followed by *A*. *viridis* genotype WAV7, while *A. spinosus* genotype WAS11 exhibited the lowest Na content. *A. viridis* genotype had higher Na content compared to *A. spinosus* genotype. Our study revealed that significant notable variations were observed in B content of *A. viridis* and *A. spinosus* genotype. *A. viridis* genotypes exhibited higher B content compared to the genotype of *A. spinosus*. Boron content ranged between 6.35 µg g^−1^ FW and 12.74 µg g^−1^ FW. Boron content was the highest in *A. viridis* genotype WAV9 and WAV713 (12.74, 12.42 µg g^−1^ FW), whereas, the lowest B content was observed in *A. spinosus* genotype WAS11, (6.35 µg g^−1^ FW). *A. viridis* genotypes exhibited higher Mo content compared to the genotype of *A. spinosus*. The Mo content had significant range of variations in the genotypes of *A. viridis* and *A. spinosus* (0.32 to 0. 42 µg g^−1^ FW). *A. viridis* genotype WAV9 had the highest Mo content (0.43 µg g^−1^ FW) followed by *A. viridis* genotype WAV7, while *A. spinosus* genotype WAS13 showed the lowest Mo content (0.32 µg g^−1^ FW). Iron, and zinc content was higher in *A. viridis* than the leaves of cassava^34^ and beach pea^35^. In this study, we observed remarkable Fe (22.19 µg g^−1^), Mn (10.23 µg g^−1^), Cu (3. 02 µg g^−1^), Zn (14.72 µg g^−1^) Na (30.26 µg g^−1^), Mo (12.74 µg g^−1^), and B (0.43 µg g^−1^) in *A. viridis* genotype (fresh weight basis). Similarly, Jimenez-Aguiar and Grusak^33^ reported high Fe, Mn, Cu, Zn, Na, Mo, and B (fresh weight basis) in different *A. spp*. including *A. viridis* and *A. spinosus*. They also stated that black nightshade, spinach, and kale had lower Zn content than amaranth; kale exhibited less Fe and Cu content than amaranth. *A. viridis* genotype had the highest Fe, Mn, Cu, Zn, Na, Mo, and B content compared to *A. spinosus* genotype.

### Antioxidant leaf pigments

Antioxidant leaf pigments of *A. viridis* and *A. spinosus* genotypes are presented in Table 4. Prominent variations in chlorophyll *a* content (267.85 to 302.56 μg g^−1^ FW) were noted in *A. viridis* and *A. spinosus* genotypes. Comparatively, *A. viridis* genotypes exhibited higher chlorophyll *a* content than *A. spinosus* genotypes. *A. viridis* genotype WAV7 showed the highest chlorophyll *a* content (302.56 μg g^−1^ FW) followed by *A. viridis* genotype WAV9. On the other hand, the lowest chlorophyll *a* content (267.85 μg g^−1^ FW) was noted in *A. spinosus* genotype WAS13 and WAS156. Similar to chlorophyll *a*, significant and noticeable differences were recorded in chlorophyll *b* content (135.26 to 152.42 μg g^−1^ FW) of selected six *A. viridis* and *A. spinosus* genotypes. *A. spinosus* genotypes showed higher chlorophyll *b* content compared to the genotype of *A. viridis*. The highest chlorophyll *b* content was observed in *A. spinosus* genotype WAS13 (152.42 μg g^−1^ FW) followed by WAS11 and WAS15. In contrast, *A. viridis* genotype WAV4 had the lowest chlorophyll *b* content (135.26 μg g^−1^ FW). The significant variations in chlorophyll *ab* content were noted in selected six *A. viridis* and *A. spinosus* genotypes. (413.61 to 445.22 μg g^−1^ FW). *A. viridis* genotypes showed higher chlorophyll *ab* content compared to the genotype of *A. spinosus*. *A. viridis* genotype WAV7 showed the highest chlorophyll *ab* content (445.22 μg g^−1^ FW) followed by *A. viridis* genotype WAV7 and *A. spinosus* genotype WAS13, while *A. spinosus* genotype WAS11 exhibited the lowest chlorophyll *ab* content (413.61 μg g^−1^ FW). In this study, we observed notable chlorophyll *a* (302.56 μg g^−1^ FW) and chlorophyll *ab* content (445.22 μg g^−1^ FW) in *A. viridis* genotype and chlorophyll *b* (152.42 μg g^−1^ FW) in *A. spinosus* genotype, whereas, Khanam and Oba^36^ reported comparatively lower chlorophyll content in red and green amaranth*. A. viridis* genotype had the highest chlorophyll *a* and chlorophyll *ab* content, while *A. spinosus* genotype exhibited the highest chlorophyll *b* content.

β-Cyanins content had no prominent variations in selected six *A. spinosus* and *A. viridis* genotypes (185.52 to 538.51 ng g^−1^ FW) albeit it showed significant variations in terms of genotypes. Comparatively, *A. viridis* genotypes exhibited higher β-cyanins content than *A. spinosus* genotypes, albeit *A. spinosus* genotype WAS15 had the highest β-cyanins content (286.46 ng g^−1^ FW) along with *A. viridis* genotypes WAV9 and WAV7 (287.56, 285.33 ng g^−1^ FW). Higher β-cyanins content was noted in *A. spinosus* genotype WAS11 (282.84 ng g^−1^ FW). On the other hand, *A. viridis* genotype WAV4 showed the lowest β-cyanins content (276.34 ng g^−1^ FW). The significant variations were observed in β-xanthins content in selected six *A. spinosus* and *A. viridis* genotypes with a range of 246.87 to 275.86 ng g^−1^ FW. *A. spinosus* genotypes exhibited higher β-xanthins content compared to *A. viridis* genotypes. *A. spinosus* genotype WAS13 exhibited the highest β-xanthins content (275.86 ng g^−1^ FW) followed by *A. spinosus* genotype WAS15. Conversely, the lowest β-xanthins content was noted in *A. viridis* genotype WAV7 (246.87 ng g^−1^ FW). The significant variations were recorded for betalains content of selected six *A. spinosus* and *A. viridis* genotypes (532.12 to 561.42 ng g^−1^ FW). *A. spinosus* genotypes exhibited higher betalains content compared to *A. viridis* genotypes. Betalains content was the highest in *A. spinosus* genotype WAS15 (561.42 ng g^−1^ FW) followed by *A. spinosus* genotype WAS13, while the lowest betalains content was reported in *A. viridis* genotype WAV4 and WAV7 (532.12, 532.24 ng g^−1^ FW). Like betalains, carotenoids showed significant variability in selected six *A. viridis* and *A. spinosus* genotypes (68.52 to 92.87 mg 100 g^−1^ FW). *A. viridis* genotypes exhibited higher carotenoids content compared to *A. spinosus* genotypes. The highest carotenoids content was observed in *A. viridis* genotype WAV7 (92.87 mg 100 g^−1^ FW) followed by *A. viridis* genotype WAV9. Whereas, *A. spinosus* genotype WAS11 and WAS13 exhibited the lowest carotenoids content (68.52, 69.82 mg 100 g^−1^ FW). Our study showed notable chlorophyll *a* (302.56 μg g^−1^ FW), chlorophyll *ab* (445.22 μg g^−1^ FW), β-cyanins (287.56 ng g^−1^ FW), and carotenoids content (92.87 mg 100 g^−1^ FW) in *A. viridis* genotype, while chlorophyll *b* (152.42 μg g^−1^ FW), β-cyanins (286.46 ng g^−1^ FW), β-xanthins (274.96 ng g^−1^ FW), and betalains content (561.42 ng g^−1^ FW) in *A. spinosus* genotype. Similarly, Khanam and Oba^36^ observed similar trend in chlorophyll *a*, chlorophyll *b*, chlorophyll *ab*, β-cyanins, β-xanthins, betalains and carotenoids content of green and red amaranth*. A. viridis* genotype had the highest chlorophyll *a*, chlorophyll *ab*, β-cyanins, and carotenoids content while *A. spinosus* genotype exhibited the highest chlorophyll *b*, β-cyanins, β-xanthins, and betalains content.

### Antioxidant phytochemicals and antioxidant capacity

β-Carotene, Vitamin C, TPC, TFC and TAC of *A. viridis* and *A. spinosus* genotypes are presented in Table 5. Pronounced variations were observed in β-carotene content of selected six *A. viridis* and *A. spinosus* genotypes which ranged from 46.76 in *A. viridis* genotype WAV7 to 64.22 mg 100 g^−1^ FW in *A. spinosus* genotype WAS11. Both species exhibited high β-carotene as compared to leafy vegetable. *A. viridis* genotypes exhibited higher β-carotene compared to *A. spinosus* genotypes. Higher β-carotene content was noticed in *A. viridis* genotype WAV4 and WAV9. *A. viridis* and *A. spinosus* genotypes showed prominent variations in vitamin C content with a range of 44.62 to 107.45 mg 100 g^−1^ FW. Both species exhibited high vitamin C as compared to leafy vegetable. Vitamin C was the highest in *A. viridis* genotype WAV7 and WAV9 (107.45, 106.64 mg 100 g^−1^ FW) and the lowest in *A. spinosus* genotype WAS11 (44.62 mg 100 g^−1^ FW). *A. viridis* genotypes exhibited higher vitamin C content compared to *A. spinosus* genotypes. Marked and significant variations were noted in total polyphenol content (TPC) of *A. viridis* and *A. spinosus* genotypes which ranged from 25.98 GAE μg g^−1^ FW to 46.72 GAE μg g^−1^ FW. Genotypes of both species exhibited high phenolics as compared to leafy vegetable. *A. viridis* genotype WAV9 showed the highest TPC content 46.72 GAE μg g^−1^ FW followed by *A. viridis* genotype WAV7. While *A. spinosus* genotype WAS13 exhibited the lowest TPC (24.98 GAE μg g^−1^ FW). TFC showed no noticeable variations in terms of six selected *A. viridis* and *A. spinosus* genotypes, though genotypes of both species had high flavonoids content (174.58 RE μg g^−1^ DW to 182.46 RE μg g^−1^ DW). *A. viridis* genotype WAV9 and *A. spinosus* genotype WAS13 had the highest TFC (182.46, 182.36 RE μg g^−1^ DW) followed by *A. spinosus* genotype WAS15, whereas *A. viridis* genotype WAV4, WAV7, and *A. spinosus* genotype WAS11 showed the lowest TFC (174.58, 175.64 and 176.46 RE μg g^−1^ DW) though both weedy species had high flavonoids. *A. spinosus* genotypes exhibited higher TFC compared to *A. viridis* genotypes, albeit differences were very low. *A. viridis* and *A. spinosus* genotypes exhibited high TAC (DPPH and ABTS^+^) as a leafy vegetable and there were pronounced variations in terms of TAC (DPPH and ABTS^+^). *A. spinosus* genotypes exhibited higher TAC (DPPH and ABTS^+^) compared to *A. viridis* genotypes. The highest TAC (DPPH and ABTS^+^) were observed in *A. spinosus* genotype WAS13 (27.56, 52.35 TEAC μg g^−1^ DW) followed by *A. spinosus* genotype WAS11 and WAS15. On the other hand, the lowest TAC (DPPH and ABTS^+^) was recorded in *A. viridis* genotype WAV7 (21.96, 48.23 TEAC μg g^−1^ DW). A similar trend of TAC (DPPH) and TAC (ABTS^+^) in terms of genotypes validated the measurement of two different methods of antioxidant capacities. The highest β-carotene, vitamin C, TPC, TFC, and TAC (DPPH and ABTS^+^) were obtained from *A. viridis* genotypes, while *A. viridis* genotypes had the highest TFC, and TAC (DPPH and ABTS^+^). In the present investigation, *A. viridis* genotypes exhibited outstanding β-carotene and vitamin C (64.22 and 107.45 mg 100 g^−1^ FW) which was higher than red amaranth of our previous studies^3^. TPC (46.72 GAE μg g^−1^ FW) obtained in this study was higher than the results of Khanam *et al*.^37^ in red and green amaranth. TFC (182.46 RE μg g^−1^ DW) TAC (DPPH) (27.56 TEAC μg g^−1^ DW) and TAC (ABTS^+^) (52.35 TEAC μg g^−1^ DW) obtained from weedy amaranth in this study, were similar to the results of Khanam *et al*.^37^ in red amaranth whereas, our obtained results were higher than the results of Khanam *et al*.^37^ in green amaranth. The *A. viridis* genotype WAV7 and *A. spinosus* genotype WAS13 had high nutrients, pigments vitamins, phenolics, flavonoids, and antioxidant. These two weedy *Amaranthus* accessions could be used as antioxidant profile enriched high-yielding varieties with high nutritional and antioxidant activity. The present investigation revealed that weedy *Amaranthus* is an excellent source of nutritional value, antioxidant phytochemicals, and antioxidant activity offered huge prospects as cultivated vegetable amaranth to feeding the mineral, vitamin, and antioxidant deficient community.

### Correlation studies

Correlation of antioxidant leaf pigments, β-carotene, vitamin C, TPC, TFC, TAC (DPPH) and TAC (ABTS^+^) of *A. viridis* and *A. spinosus* genotypes are presented in Table 6. Correlation of antioxidant leaf pigments, β-carotene, vitamin C, TPC, TFC, TAC (DPPH) and TAC (ABTS^+^) of *A. viridis* and *A. spinosus* genotypes showed interesting results. Significant positive associations with TPC, TFC, TAC (DPPH) and TAC (ABTS^+^) were observed for all antioxidant leaf pigments. It indicated that the increase in TPC, TFC, TAC (DPPH) and TAC (ABTS^+^) were directly related to the increment of chlorophylls, β-cyanins, β-xanthins, betalains, and carotenoids content or vice versa. It meant all leaf pigments had strong antioxidant activity. Similarly, vitamin C had a significant positive interrelationship with TPC, TFC, and TAC, while it exhibited insignificant negative associations among all antioxidant leaf pigments. Sarker and Oba^18,24^ in their earlier work in amaranth also observed a similar trend. A significant positive association was exhibited among β-carotene, vitamin C, TPC, TFC, TAC (DPPH), and TAC (ABTS^+^). The significant positive interrelationship of β-carotene, vitamin C, TPC, TFC, TAC (DPPH), and TAC (ABTS^+^) signify that β-carotene, vitamin C, TPC, TFC had strong antioxidant activity. The validation of the antioxidant capacity of *A. viridis* and *A. spinosus* genotypes by two different methods of antioxidant capacity measurements were confirmed with the significant positive associations between TAC (DPPH) and TAC (ABTS^+^). Antioxidant phytochemicals such as leaf pigments, β-carotene, vitamin C, TPC, and TFC had strong antioxidant activity, as these showed the significant associations with TAC (DPPH) and TAC (ABTS^+^). In the present investigation, all antioxidant leaf pigments, β-carotene, vitamin C, TPC, and TFC played a vital role in the antioxidant activity of *A. viridis* and *A. spinosus* genotypes as these compounds had strong antioxidant activity.

**Table 6:** Correlation coefficient of antioxidant leaf pigments, vitamin C, TPC, TFC, TAC (DPPH) and TAC (ABTS^+^) of six selected genotypes of *A. viridis* and *A. spinosus* weedy species.

Traits	Chl *b* (µg g^−1^ FW)	Chl *ab* (µg g^−1^ FW)	β-cyanins (ng g^−1^ FW)	β-xanthins (ng g^−1^ FW)	Betalains (ng g^−1^ FW)	Carotenoids (mg 100 g^−1^ FW)	β-Carotene (mg 100 g^−1^ FW)	Vitamin C (mg 100 g^−1^ FW)	TPC (GAE µg g^−1^ FW)	TFC (RE µg g^−1^ DW)	TAC (DPPH) (TEAC µg g^−1^ DW)	TAC (ABTS^+^) (TEAC µg g^−1^ DW)
Chlorophyll *a* (µg g^−1^ FW)	0.87**	0.96**	0.86**	0.88**	0.88**	−0.82*	−0.71*	−0.017	0.88**	0.87**	0.89**	0.95**
Chlorophyll *b* (µg g^−1^ FW)		0.93**	0.72*	0.86**	0.85**	−0.76	−0.64	−0.028	0.74*	0.74*	0.88**	0.89**
Chlorophyll *ab* (µg g^−1^ FW)			0.72*	0.75*	0.84**	−0.87*	−0.76*	−0.023	0.77*	0.79**	0.77*	0.88**
β-cyanins (ng g^−1^ FW)				0.89**	0.95**	−0.86*	−0.77*	−0.112	0.76*	0.786**	0.94**	0.89**
β-xanthins (ng g^−1^ FW)					0.98**	−0.88**	−0.87**	−0.132	0.72*	0.73*	0.76*	0.96**
Betalains (ng g^−1^ FW)						−0.92**	−0.92**	−0.125	0.94**	0.77*	0.75**	0.98**
Carotenoids (mg 100 g^−1^ FW)							0.86**	−0.232	0.95**	0.89**	0.96**	0.95**
β-Carotene (mg 100 g^−1^ FW)								0.76*	0.82**	0.95**	0.87**	0.88**
Vitamin C (mg 100 g^−1^ FW)									0.75*	0.92**	0.89**	0.98**
TPC (GAE µg g^−1^ FW)										0.88**	0.88**	0.97**
TFC (RE µg g^−1^ DW)											0.86**	0.96**
TAC (DPPH) (TEAC µg g^−1^ DW)												0.95**

Chl *a*, Chlorophyll *a;* Chl *ab*, Chlorophyll *ab;* TAC, Total antioxidant capacity; TPC, Total polyphenol content; TFC, Total flavonoid content; *Significant at 5% level, **Significant at 1% level.

In conclusion, the present study has demonstrated that leaves of both weedy *Amaranthus* genotypes exhibited as a good source of potassium, calcium, magnesium, P, S, Fe, Mn, Cu, Zn, Na, B, Mo, protein, dietary fiber, carbohydrates as a leafy vegetable. It is an excellent source of antioxidant leaf pigments, β-carotene, vitamin C, TAC, TPC and TFC and antioxidant that could contribute to human nutrition and health. The *A. viridis* genotype WAV7 and *A. spinosus* genotype WAS13 identified as the best accessions and could be cultivated as like as cultivar as a potential source of nutritional value, antioxidant leaf pigments, β-carotene, vitamin C, phenolics, flavonoids and antioxidants in our daily diet to reduce the hidden hunger and accomplishing nutritional and antioxidant sufficiency. Weedy *Amaranthus* species are the excellent source of phenolics, flavonoids, and antioxidants that have many pharmacological effects of their traditional applications. Finally, the obtained data present a valuable contribution to the scientific evaluation of pharmacologically active principles in weedy species.

## Methods

### Experiment materials, design, layout, and cultural practices

Department of Genetics and Plant Breeding of Bangabandhu Sheikh Mujibur Rahman Agricultural University collected several accessions (genotypes) of weedy amaranth *(A. spinosus* and *A. viridis*) from different agro-ecological zones of Bangladesh. We selected six genotypes (three accessions from each species) based on different morphological traits and different agroecological zones. We grew the selected genotypes at the experimental field of Bangabandhu Sheikh Mujibur Rahman Agricultural University, Bangladesh in a randomized complete block design (RCBD) with three replications. The unit plot size of each genotype was one square meter. The spacing of each *A. spinosus* and *A. viridis* genotype was 20 cm distance from row to row and 5 cm distance from the plant to plant. Recommended fertilizer, compost doses, and appropriate cultural practices were maintained. Thinning was done to maintain appropriate spacing between plants of a row. As a necessity, weeding and hoeing were done to remove the weed. To maintain the normal growth of the crop proper irrigations were provided. At 30 days after sowing of seed, leaves samples were collected. All the parameters were measured in three replicates.

### Chemicals

Solvent: acetone and methanol. Reagents: H_2_SO_4_, HNO_3_, HClO_3,_ NaOH, dithiothreitol (DTT), caesium chloride, ascorbic acid, standard compounds of pure Trolox (6-hydroxy-2, 5, 7, 8-tetramethyl-chroman-2-carboxylic acid), gallic acid, rutin, folin-ciocalteu reagent, DPPH (2, 2-diphenyl1-picrylhydrazyl), ABTS^+^, aluminium chloride hexahydrate, sodium carbonate, potassium acetate, and potassium persulfate. All solvents and reagents used in this study were high purity laboratory products obtained from Kanto Chemical Co. Inc. (Tokyo, Japan) and Merck (Germany).

### Proximate composition

AOAC method was followed^18^ to estimate the ash, moisture, crude fat, fiber, crude protein contents, and gross energy. Micro-Kjeldahl method was followed to determine crude protein with nitrogen to a protein conversion factor of 6.25 (AOAC method 976.05). We subtracted the sum of moisture, ash, crude fat, and crude protein percentage from 100 to measure carbohydrate content (g 100 g^−1^ FW).

### Estimation of mineral content

At first, *A. spinosus* and *A. viridis* leaves were dried at 70 °C in a well-ventilated oven for 24 hours. Dried leaves were grounded finely in a mill. Nitric-perchloric acid digestion method^18^ was followed to determine the macronutrients (Ca, Mg, K, P, and S) and microelements (Fe, Mn, Cu, Zn, Na, Mo, and B) from powdered leaves. For this digestion, 400 ml of nitric acid (65%), 40 ml of perchloric acid (70%) and 10 ml of sulphuric acid (96%) in the presence of carborundum beads were added to 0.5 g dried leaf sample. After digestion, the solution was appropriately diluted in triplicate for measuring P following ascorbic acid method. Yellow-colored complex converted to a blue-colored phosphomolybdenum complex when ascorbic acid and Sb was added to the solution. Sarker and Oba^18^ method was followed to read the absorbance by atomic absorption spectrophotometry (AAS) (Hitachi, Tokyo, Japan) at wavelength of 76 6.5 nm (K), 422.7 nm (Ca), 285.2 nm (Mg), 880 nm (P), 258.056 nm (S), 248.3 nm (Fe), 279.5 nm (Mn), 324.8 nm (Cu), 213.9 nm (Zn), 589 nm (Na), 313.3 nm (Mo), and 430 nm (B).

### Determination of chlorophylls and total carotenoids

The fresh *A. spinosus* and *A. viridis* leaves were extracted in 80% acetone to estimate chlorophyll *a*, chlorophyll *b*, chlorophyll *ab* and total carotenoids following Sarker and Oba^18^ method. A spectrophotometer (Hitachi, U-1800, Tokyo, Japan) was used to read the absorbance at 663, 646 and 470 nm for chlorophyll *a*, chlorophyll *b* and total carotenoids, respectively. Data were expressed as μg chlorophyll per g fresh weight (FW) and mg carotenoids per 100 g FW.

### Determination of β-cyanin and β-xanthin content

The fresh *A. spinosus* and *A. viridis* leaves were extracted in 80% methanol containing 50 mM ascorbic acid to measure β-cyanin and β-xanthin following the method of Sarker and Oba^18^. A spectrophotometer (Hitachi, U-1800, Tokyo, Japan) was used to read the absorbance at 540 and 475 nm for β-cyanin and β-xanthin, respectively. The results were expressed as nanogram betanin equivalent to per gram FW for β-cyanin and nanograms indicaxanthin equivalent to per gram FW for β-xanthin.

### Estimation of β-carotene

Method of Sarker and Oba^18^,^38^ was followed to extract and determine β-carotene content. 500 mg of fresh leaf sample was ground in 10 ml of 80% acetone and centrifuged at 10,000 rpm for 3–4 min to carry out the extraction process. The final volume was brought up to 20 ml after removing the supernatant in a volumetric flask. A spectrophotometer (Hitachi, U-1800, Tokyo, Japan) was used to read the absorbance at 510 nm and 480 nm. Data were expressed as mg β-carotene per 100 g fresh weight.

The β-carotene content was calculated using the following formula:

$${\rm{Amount}}\,{\rm{of}}\,{\rm{\beta }} \mbox{-} \mathrm{carotene}={\rm{7.6}}\,({\rm{Abs}}{\rm{.}}\,{\rm{at}}\,{\rm{480}})-{\rm{1.49}}({\rm{Abs}}{\rm{.}}\,{\rm{at}}\,{\rm{510}})\times {\rm{Final}}\,{\rm{volume}}/({\rm{1000}}\times {\rm{fresh}}\,{\rm{weight}}\,{\rm{of}}\,{\rm{leaf}}\,{\rm{taken}})$$

### Determination of vitamin C

The fresh *A. spinosus* and *A. viridis* leaves were used to measure ascorbic acid (AsA) and dehydroascorbate (DHA) acid spectrophotometrically. For pre-incubation of the sample and reduction of DHA into AsA Dithiothreitol (DTT) was used. AsA reduced Fe_3_^+^ to Fe_2_^+^ and estimation of AsA was made by the spectrophotometric (Hitachi, U-1800, Tokyo, Japan) measuring Fe_2_^+^ complexes with 2, 2-dipyridyl^18^,^39^. Finally, the absorbance of the sample solution was read. Data were recorded as mg ascorbic acid per 100 g fresh weight (FW).

### Extraction of samples for TPC, TFC and TAC analysis

At the edible stage (30 Days after sowing), *A. spinosus* and *A. viridis* leaves were harvested. The leaves were air dried in shade for chemical analysis. 40 ml of 90% aqueous methanol was used to extract 1 g of grounded dried leaves from each cultivar in a tightly capped bottle (100 ml). The extract was then placed in a shaking water bath (Thomastant T-N22S, Thomas Kagaku Co. Ltd., Japan) for 1 h. Then the extract was filtered for further analytical assays of total polyphenol content, total flavonoid content, total antioxidant activity.

### Determination of total polyphenol content (TPC)

Method of Sarker and Oba^18^,^40^ was followed to estimate the total phenolic content of *A. spinosus* and *A. viridis* using the folin-ciocalteu reagent with gallic acid as a standard phenolic compound. In a test tube, 1 ml of folin-ciocalteu reagent (previously diluted 1:4, reagent: distilled water) was added to 50 µl of the leaf extract solution and then mixed thoroughly for 3 min. Then, the mixture was allowed to stand for 1 h in the dark by adding 1 ml of Na_2_CO_3_ (10%). A Hitachi U1800 spectrophotometer (Hitachi, Tokyo, Japan) was used to read the absorbance was read at 760 nm. An equation obtained from a standard gallic acid graph was used to estimate the concentration of total phenolic compounds in the leaf extracts. The results are expressed as μg gallic acid equivalent (GAE) g^−1^ FW.

### Determination of total flavonoid content (TFC)

The aluminum chloride colorimetric method^38^,^41^ was used to estimate the total flavonoid content of *A. spinosus* and *A. viridis* extract. In a test tube, 1.5 ml of methanol was added to 0.1 ml of 10% aluminum chloride, 0.1 ml of 1 M potassium acetate, 2.8 ml of distilled water and 500 µl of leaf extract for 30 min at room temperature. A Hitachi U1800 spectrophotometer (Hitachi, Tokyo, Japan) was used to take the absorbance of the reaction mixture at 415 nm. TFC is expressed as μg rutin equivalent (RE) g^−1^ dry weight (DW) using rutin as the standard compound.

### Total antioxidant capacity (TAC)

Diphenyl-picrylhydrazyl (DPPH) radical degradation method^39^,^42^ was used to estimate the antioxidant activity. In a test tube, 1 ml of 250 µM DPPH solution was added to 10 µl of leaf extract solution (in triplicate) and 4 ml of distilled water and allowed to stand for 30 min in the dark. A Hitachi U1800 spectrophotometer (Hitachi, Tokyo, Japan) was used to read the absorbance at 517 nm. Method of Sarker and Oba^39^,^43^was followed for ABTS^+^ assay. 7.4 mM ABTS^+^ solution and 2.6 mM potassium persulfate were used in the stock solutions. The two stock solutions were mixed in equal quantities and allowing them to react for 12 h at room temperature in the dark for preparation of the working solution. 2850 μl of ABTS^+^ solution (1 ml ABTS^+^ solution mixed with 60 ml methanol) was mixed with 150 μl sample of leaf extract and allowed to react for 2 h in the dark. Aa Hitachi U1800 spectrophotometer (Hitachi, Tokyo, Japan) was used to read the absorbance against methanol at 734 nm. The percent of inhibition of DPPH and ABTS^+^ relative to the control were used to determine antioxidant activity using the following equation:

$${\rm{Antioxidant}}\,{\rm{activity}}( \% )=({\rm{Abs}}{\rm{.}}\,{\rm{blank}}-{\rm{Abs}}{\rm{.}}\,{\rm{sample}}/{\rm{Abs}}{\rm{.}}\,{\rm{blank}})\times {\rm{100}}$$

where, Abs. blank is the absorbance of the control reaction [10 µl methanol for TAC (DPPH), 150 μl methanol for TAC (ABTS^+^) instead of leaf extract] and Abs. sample is the absorbance of the test compound. Trolox was used as the reference standard, and the results were expressed as μg Trolox equivalent g^−1^ DW.

### Statistical analysis

The results were reported as the average of three measurements (n = 3). The data were also statistically analyzed by ANOVA using Statistix 8 software, and the means were compared by the Duncan’s Multiple Range Test (DMRT) at 1% and level of probability.

### Ethical statement

The lab and field experiment in this study was carried out following guidelines and recommendations of “Biosafety Guidelines of Bangladesh” published by Ministry of Environment and Forest, Government of the People’s Republic of Bangladesh (2005).

## Data Availability

Data used in this manuscript will be available to the public.
